# Natural convection heat transfer of nanofluids along a vertical plate embedded in porous medium

**DOI:** 10.1186/1556-276X-8-64

**Published:** 2013-02-07

**Authors:** Ziya Uddin, Souad Harmand

**Affiliations:** 1 Université de Lille Nord de France, Lille, F-59000, France; 2 TEMPO/DF2T, UVHC, Valenciennes, F-59313, France

**Keywords:** Nanofluid, Convection, Porous medium, Implicit FDM, Numerical solution

## Abstract

The unsteady natural convection heat transfer of nanofluid along a vertical plate embedded in porous medium is investigated. The Darcy-Forchheimer model is used to formulate the problem. Thermal conductivity and viscosity models based on a wide range of experimental data of nanofluids and incorporating the velocity-slip effect of the nanoparticle with respect to the base fluid, i.e., Brownian diffusion is used. The effective thermal conductivity of nanofluid in porous media is calculated using copper powder as porous media. The nonlinear governing equations are solved using an unconditionally stable implicit finite difference scheme. In this study, six different types of nanofluids have been compared with respect to the heat transfer enhancement, and the effects of particle concentration, particle size, temperature of the plate, and porosity of the medium on the heat transfer enhancement and skin friction coefficient have been studied in detail. It is found that heat transfer rate increases with the increase in particle concentration up to an optimal level, but on the further increase in particle concentration, the heat transfer rate decreases. For a particular value of particle concentration, small-sized particles enhance the heat transfer rates. On the other hand, skin friction coefficients always increase with the increase in particle concentration and decrease in nanoparticle size.

## Background

Natural convection heat transfer in porous media is an important phenomenon in engineering systems due to its wide applications such as cooling of electronics components, heat exchangers, drying processes, building insulations, and geothermal and oil recovery. Due to high surface area, fluid mixing qualities, high thermal conductivity, and wide industrial applications, natural convection through porous media has gained considerable attention of various researchers in the past few decades. Cheng and Minkowycz [1] studied free convection about a vertical flat plate embedded in a porous medium with application to heat transfer from a dike. They used the boundary layer approximations and found the similarity solution for the problem. Evans and Plumb [2] investigated natural convection about a vertical plate embedded in a medium composed of glass beads with diameters ranging from 0.85 to 1.68 mm. Their experimental data was in good agreement with the theory. Cheng [3] and Hsu [4] investigated the Darcian free convection flow about a semi-infinite vertical plate. They used the higher-order approximation theory and confirmed the results of Evans and Plumb [2]. Kim and Vafai [5] analyzed the natural convection about a vertical plate embedded in a porous medium. They took two cases in their analysis, *viz*., constant wall temperature and constant heat flux. They found the analytic solution for the boundary layer flow using the methods of matching asymptotes. Badruddin et al. [6] investigated free convection and radiation for a vertical wall with varying temperatures embedded in a porous medium. Steady and unsteady free convection in a fluid past an inclined plate and immersed in a porous medium was studied by Chamka et al. [7] and Uddin and Kumar [8]. They used the Brinkmann-Forchheimer model for the flow in porous media. Some more details about the theoretical and experimental studies for the convection in porous media can be found in the work of Neild and Bejan [9].

In industries, heat transfer can be enhanced by modifying the design of the devices, e.g., increasing the surface area by addition of fins, applying magnetic field and electric field. In compact-designed devices, these techniques are hard to apply, so the other option for heat transfer enhancement is to use the fluid with high thermal conductivity. However, common fluids like water, ethylene glycol, and oil have low values of thermal conductivities. On the other hand, the metals and their oxide have high thermal conductivities compared to these fluids. Choi [10] proposed that the uniform dispersion of small concentration of nano-sized metal/metal oxides particles into a fluid enhances the thermal conductivity of the base fluid, and such fluids were termed as nanofluids. This concept attracted various researchers towards nanofluids, and various theoretical and experimental studies have been done to find the thermal properties of nanofluids. An extensive review of thermal properties of nanofluids can be found in the study of Wang and Majumdar [11]. From the literature, it is found that the thermal conductivity of nanofluids depends upon various factors, such as particle material, base fluid material, particle volume fraction, particle size, particle shape, temperature, nanoparticle Brownian motion, nanoparticle base fluid interfacial layer, and particle clustering. It is also found in the study of Wang and Majumdar [12] that the insertion of nanoparticle increases the viscosity of the fluid. There are various theoretical relations predicting the thermal conductivity and viscosity of nanofluids, but these empirical relations do not satisfy the experimental data up to a satisfying range. Chon et al. [13] found an empirical correlation for the thermal conductivity of nanofluids within the particle size range of 11 to 150 nm and temperature range of 21°C to 71°C. They reported that the Brownian motion of nanoparticles constitutes a key mechanism of the thermal conductivity enhancement with increasing temperature and decreasing nanoparticle sizes. However, this empirical formula was valid only for water-Al_2_O_3_ nanofluid. Very recently, Corcione [14] analyzed the experimental data of thermal conductivity and viscosity of nanofluids, which were obtained by various researchers for different types of nanoparticles dispersed in different base fluids, and found an empirical correlating equation for the prediction of effective thermal conductivity and dynamic viscosity of nanofluids.

With the advances in thermal properties and viscosity of nanofluids, various researchers studied the convective flow numerically as well as experimentally. Ho et al. [15] studied the natural convection of nanofluid having a particle concentration within the range of 0% to 4% in a square enclosure and analyzed the effects caused by uncertainties of viscosity and thermal conductivity. This study was limited to Al_2_O_3_-water nanofluid only. A detailed study of the natural convection of water-based nanofluids in an inclined enclosure has been done by Elif [16]. In this study, he investigated heat transfer enhancement using five different types of nanoparticles dispersed in water. To model the problem, he used a renovated Maxwell model containing the effect of interfacial layers in the enhanced thermal conductivity of nanofluids, given by Yu and Choi [17]. Abu-Nada and Oztop [18] investigated the effects of inclination angle on natural convection in enclosures filled with Cu-water nanofluid. All these authors reported that the heat transfer rate increases with the increase in nanoparticle concentration in the base fluid. However, in these studies, the effect of temperature and Brownian motion was not considered in the formulation of the problem. Abu-Nada [19] investigated the natural convection heat transfer in horizontal cylindrical annulus filled with Al_2_O_3_-water nanofluid taking the effect of variable viscosity and thermal conductivity. In the study, the effective thermal conductivity was calculated by the model of Chon et al. [13], and to formulate the dynamic viscosity of the Al_2_O_3_-water nanofluid, the author used the experimental data and found the empirical correlation for the dynamic viscosity as a function of temperature and particle concentration. Ho et al. [20] investigated the natural convection heat transfer of alumina-water nanofluid in vertical square enclosure experimentally. They reported that on higher Rayleigh numbers, the heat transfer rate increases on the dispersion of very small quantity of nanoparticles in water, but a larger quantity of nanoparticles in water decreases the heat transfer rates. The natural convection of nanofluids past vertical plate under different conditions has been studied by Hamad and Pope [21] and Rana and Bhargava [22]. They reported that the Nusselt number as well as the skin friction coefficient both increase with the increase in nanoparticle concentration in the base fluid. Zoubida et al. [23] investigated the effects of thermophoresis and Brownian motion significant in nanofluid heat transfer enhancement and found an enhancement in heat transfer at any volume fraction of nanoparticles. They also reported that the enhancement is more pronounced at low volume fraction of nanoparticles and that the heat transfer decreases by increasing the nanoparticle volume fraction.

The dispersion of nano-sized particles in the traditional fluid increased the thermal conductivity of the fluid, and the presence of porous media enhances the effective thermal conductivity of the base fluid. Thus, the use of nanofluids in porous media would be very much helpful in heat transfer enhancement. So far, very few studies have been done for the natural convection of nanofluids in porous media. Nield and Kuznetsov [24] studied the Cheng-Minkowycz problem for natural convection boundary layer flow in a porous medium saturated by a nanofluid. In the modeling of the problem, they used nanofluids by incorporating the effects of Brownian motion and thermophoresis. For the porous medium, the Darcy model was taken. Aziz et al. [25] found the numerical solution for the free convection boundary layer flow past a horizontal flat plate embedded in porous medium filled by nanofluid containing gyrotactic microorganisms. Recently, Rana et al. [26] found the numerical solution for steady-mixed convection boundary layer flow of a nanofluid along an inclined plate embedded in a porous medium. In the studies of natural convection of nanofluids in porous media, the authors did the parametric study only. However, they did not account any effect of parameters influencing the thermal conductivity and dynamic viscosity, such as particle concentration, particle size, temperature, nature of base fluid, and the nature of nanoparticle, which satisfy the experimental data for the thermal conductivity and dynamic viscosity of the nanofluids. In the best knowledge of the authors of this article, no such study has been done with regard to the natural convection of nanofluids in porous media. It is known that heat transfer in a fluid depends upon the temperature difference in fluid and heated surface and the thermophysical properties of the fluid. Heat transfer also depends upon the fluid flow rate, which depends upon the viscosity of the fluid.

As seen from the literature, most of the experimental studies on the thermal properties of nanofluids proved that the thermal conductivity of nanofluid depends upon the nanoparticle material, base fluid material, particle volume concentration, particle size, temperature, and nanoparticle Brownian motion. In previous works related to the flow of nanofluid in porous media, the authors used the variable thermophysical properties of the nanofluids, but it did not satisfy the experimental data for a wide range of reasons. Also, they did not consider the heat transfer through the two phases, i.e., nanofluid and porous media.

Therefore, the scope of the current research is to implement the appropriate models for the nanofluid properties, which consist the velocity-slip effects of nanoparticles with respect to the base fluid and the heat transfer flow in the two phases, i.e., through porous medium and nanofluid to be taken into account, and to analyze the effect of nanofluids on heat transfer enhancement in the natural convection in porous media.

## Methods

### Mathematical formulation

**Figure 1:**
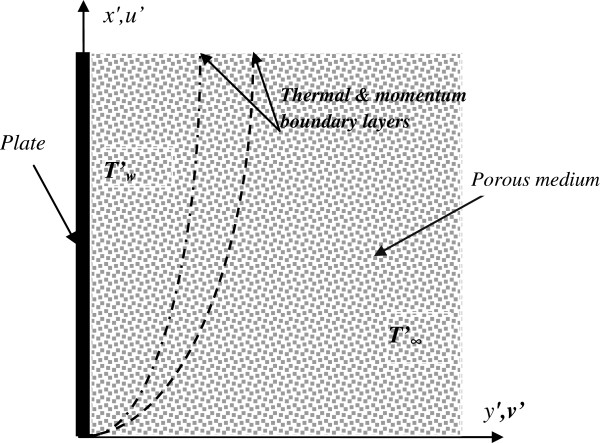
Physical model and coordinate system.

A problem of unsteady, laminar free convection flow of nanofluids past a vertical plate in porous medium is considered. The *x*-axis is taken along the plate, and the *y*-axis is perpendicular to the plate. Initially, the temperature of the fluid and the plate is assumed to be the same. At *t*^′^ > 0, the temperature of the plate is raised to *T*_*w*_^'^, which is then maintained constant. The temperature of the fluid far away from the plate is *T*_*∞*_^'^. The physical model and coordinate system are shown in Figure 1.

The Brinkman-Forchheimer model is used to describe the flow in porous media with large porosity. Under Boussinesq approximations, the continuity, momentum, and energy equations are as follows:

(1) $$\frac{\partial u'}{\partial x'}+\frac{\partial v'}{\partial y'}=0$$

(2) $$\begin{array}{c} \frac{\partial u'}{\partial t'}+u'\frac{\partial u'}{\partial x'}+v'\frac{\partial u'}{\partial y'}=g\beta_{\mathrm{nf}}\left(T'-T_{\infty }^{'}\right)\\ \;+\frac{\mu_{\mathrm{nf}}}{\rho_{\mathrm{nf}}}\frac{\partial^{2}u'}{\partial y{'}^{2}}-\frac{\mu_{\mathrm{nf}}}{\rho_{\mathrm{nf}}}\frac{\varepsilon }{K}u'\\ \;-\frac{F\varepsilon^{2}}{K^{1/2}}u'\left|u'\right|, \end{array}$$

(3) $$\begin{array}{c} \sigma \frac{\partial T'}{\partial t'}+u'\frac{\partial T'}{\partial x'}+v'\frac{\partial T'}{\partial y'}=\frac{\partial }{\partial y'}\left(\alpha_{\mathrm{eff}}\frac{\partial T'}{\partial y'}\right)-\frac{u'}{C_{p_{\mathrm{nf}}}}\\ \;\times \left[\frac{\mu_{\mathrm{nf}}}{\rho_{\mathrm{nf}}}\frac{\partial^{2}u'}{\partial y{'}^{2}}-\frac{\mu_{\mathrm{nf}}}{\rho_{\mathrm{nf}}}\frac{\varepsilon }{K}u'-\frac{F\varepsilon^{2}}{K^{1/2}}u'\left|u'\right|\right]. \end{array}$$

Here, *u*^′^ and *v*^′^ are the velocity components along the *x*^′^ and *y*^′^ axes. *T*^′^ is the temperature inside the boundary layer, *ε* is the porosity of the medium, *K* is the permeability of porous medium, and *F* is the Forchheimer constant.

The quantities with subscript ‘nf’ are the thermophysical properties of nanofluids, *α*_eff_ is the effective thermal diffusivity of the nanofluid in porous media, and *σ* is the volumetric heat capacity ratio of the medium. These quantities are defined as follows:

(4) $$\rho_{\mathrm{nf}}=\phi \rho_{p}+\left(1-\phi \right)\rho_{f},$$

(5) $${\left(\rho \beta \right)}_{\mathrm{nf}}=\phi {\left(\rho \beta \right)}_{p}+\left(1-\phi \right){\left(\rho \beta \right)}_{f},$$

(6) $${\left(\rho C_{p}\right)}_{\mathrm{nf}}=\phi {\left(\rho C_{p}\right)}_{p}+\left(1-\phi \right){\left(\rho C_{p}\right)}_{f},$$

(7) $$\alpha_{\mathrm{eff}}=\frac{k_{\mathrm{eff}}}{{\left(\rho C_{p}\right)}_{\mathrm{nf}}},$$

(8) $$\sigma =\frac{\varepsilon {\left(\rho C_{p}\right)}_{\mathrm{nf}}+\left(1-\varepsilon \right){\left(\rho C_{p}\right)}_{m}}{{\left(\rho C_{p}\right)}_{\mathrm{nf}}}.$$

Since the heat transfer is through the nanofluid in porous media, the effective thermal conductivity in the two phases is given as follows:

(9) $$k_{\mathrm{eff}}=\frac{k_{s}k_{\mathrm{nf}}}{\varepsilon k_{s}+\left(1-\varepsilon \right)k_{\mathrm{nf}}},$$

Here, *k*_*s*_ is the thermal conductivity of the porous material, and *k*_nf_ is the thermal conductivity of the nanofluid. Taking various physical factors into account and using a wide range of experimental data, Corcione [14] suggested an empirical correlation for the thermal conductivity of the nanofluids with particle diameters ranging from 10 to 150 nm, which is given in the following equation:

(10) knfknf=1+4.4Re0.4Pr0.66T'Tfr10kpkf0.03ϕ0.66

Here, Re is the nanoparticle Reynolds number, Pr is the Prandtl number of base fluid, *T*_fr_ is the freezing point of the base fluid, *T′* is the nanofluid temperature. Re and Pr are defined as follows:

Re=ρfuBdpμf,Pr=μfCpfkf

The mean Brownian velocity *u*_*B*_ is given by:

$$u_{B}=\frac{2k_{b}T'}{\pi \mu_{f}d_{p}^{2}}$$

Here, *k*_*b*_ is the Boltzmann's constant. Following Corcione [14], the viscosity of nanofluid is given as follows:

(11) $$\mu_{\mathrm{nf}}=\frac{\mu_{f}}{1-34.87{\left(d_{p}/d_{f}\right)}^{-0.3}\phi^{1.03}},$$

$$d_{f}=0.1{\left[\frac{6M}{\mathrm{N\pi}\rho_{\mathrm{fo}}}\right]}^{1/3}.$$

Here, *d*_*f*_ is the diameter of base fluid molecule, *M* is the molecular weight of the base fluid, *N* is the Avogadro number, and *ρ*_fo_ is the mass density of the base fluid calculated at the reference temperature.

In this model, it is assumed that the vertical plate is at uniform temperature (*T*_*w*_ '), and the lower end of the plate is at ambient temperature (*T*_*∞*_ '). Therefore, the initial and boundary conditions for the flow are as follows:

$$t\prime \leq 0:u\prime =0,v\prime =0;\;T\prime =T_{\infty }\prime \;\mathrm{for}\;x\prime \mathrm{and}\;y\prime ,$$

(12) $$t\prime >0:\{\begin{array}{l} u\prime =0,v\prime =0;\;T\prime =T_{\infty }\prime \;\mathrm{at}\;x\prime =0.\\ u\prime =0,v\prime =0;\;T\prime =T_{w}\prime \;\mathrm{at}\;y\prime =0,\;x\prime >0,\;\\ u\prime =0,\;T\prime =T_{\infty }\prime \;\mathrm{at}\;y\prime \to \infty \;x\prime >0. \end{array}$$

To simplify Equations 1, 2, and 3 along with the boundary conditions (Equation 12), following nondimensional quantities are introduced.

(13) $$\begin{array}{c} x=\frac{x'}{L},y=\frac{y'}{L},t=\frac{\alpha_{f}}{L^{2}}t',u=\frac{u'L}{\alpha_{f}},v=\frac{v'L}{\alpha_{f}},\\ \theta =\frac{T'-T_{\infty }^{'}}{T_{w}^{'}-T_{\infty }^{'}}\cdot \alpha_{f}=\frac{k_{f}}{{\left(\rho C_{p}\right)}_{f}} \end{array}$$

Therefore, the transformed equations are as follows:

(14) $$\frac{\partial u}{\partial x}+\frac{\partial v}{\partial y}=0,$$

(15) $$\begin{array}{c} \frac{\partial u}{\partial t}+u\frac{\partial u}{\partial x}+v\frac{\partial u}{\partial y}=\beta_{c}\mathrm{RaPr}\theta +\mathrm{Pr}\frac{\mu_{c}}{\rho_{c}}\frac{\partial^{2}u}{\partial y^{2}}\\ \;-\frac{\mathrm{Pr}}{\mathrm{Da}}\frac{\mu_{c}}{\rho_{c}}u-\frac{\mathrm{Fr}}{\mathrm{Da}}u\left|u\right|, \end{array}$$

$$\begin{array}{c} \sigma \frac{\partial \theta }{\partial t}+u\frac{\partial \theta }{\partial x}+v\frac{\partial \theta }{\partial y}=\frac{k_{\mathrm{eff}}\left(\theta \right)}{\alpha_{f}{\left(\rho C_{p}\right)}_{\mathrm{nf}}}\frac{\partial^{2}\theta }{\partial y^{2}}\\ \;+\frac{1}{\alpha_{f}{\left(\rho C_{p}\right)}_{\mathrm{nf}}}\frac{\partial k_{\mathrm{eff}}\left(\theta \right)}{\partial y}\frac{\partial \theta }{\partial y}\\ \;-\frac{\mathrm{Ec}.u}{{\left(C_{p}\right)}_{c}}\left[\mathrm{Pr}\frac{\mu_{c}}{\rho_{c}}\frac{\partial^{2}u}{\partial y^{2}}-\frac{\mu_{c}}{\rho_{c}}\frac{\mathrm{Pr}}{\mathrm{Da}}u-\frac{\mathrm{Fr}}{\mathrm{Da}}u\left|u\right|\right], \end{array}$$

or

(16) $$\begin{array}{c} \sigma \frac{\partial \theta }{\partial t}+u\frac{\partial \theta }{\partial x}+v\frac{\partial \theta }{\partial y}=\frac{k_{\mathrm{eff}}\left(\theta \right)}{a_{f}{\left(\rho C_{p}\right)}_{\mathrm{nf}}}\frac{\partial^{2}\theta }{\partial y^{2}}\\ \;+\frac{1}{\alpha_{f}{\left(\rho C_{p}\right)}_{\mathrm{nf}}}A\left(\theta \right){\left(\frac{\partial \theta }{\partial y}\right)}^{2}\\ \;-\frac{\mathrm{Ec}.u}{{\left(C_{p}\right)}_{c}}\left[\mathrm{Pr}\frac{\mu_{c}}{\rho_{c}}\frac{\partial^{2}u}{\partial y^{2}}-\frac{\mu_{c}}{\rho_{c}}\frac{\mathrm{Pr}}{\mathrm{Da}}u-\frac{\mathrm{Fr}}{\mathrm{Da}}u\left|u\right|\right]. \end{array}$$

The function *A*(*θ*) can be found using Equations 9 and 10. The nondimensional constants, Eckert number (Ec), Rayleigh number (Ra), Forchheimer's coefficient (Fr), and Darcy number (Da) are given as follows:

$$\begin{array}{c} \mathrm{Ec}=\frac{\alpha_{f}^{2}}{L^{2}{\left(C_{p}\right)}_{f}\left(T_{w}^{\prime }-T_{\infty }^{\prime }\right)},\mathrm{Ra}=\frac{g\rho_{f}\beta_{f}\left(T_{w}^{\prime }-T_{\infty }^{\prime }\right)L^{3}}{\alpha_{f}\mu_{f}},\\ \mathrm{Fr}=\frac{\mathrm{F\varepsilon}K^{1/2}}{L},\mathrm{Da}=\frac{K}{\varepsilon L^{2}}, \end{array}$$

The other nondimensional coefficients appeared in Equations 15 and 16 and are given as follows:

$$\begin{array}{c} \beta_{c}=\frac{\phi \rho_{p}\left(\beta_{p}/\beta_{f}\right)+\left(1-\phi \right)\rho_{f}}{\rho_{\mathrm{nf}}},\\ \rho_{c}=\phi \left(\rho_{p}/\rho_{f}\right)+\left(1-\phi \right)\\ \;\mu_{c}=\frac{1}{1-34.87{\left(d_{p}/d_{f}\right)}^{-0.3}\phi^{1.03}},\\ {\left(C_{p}\right)}_{c}=\frac{\phi \rho_{p}\left[{\left(C_{p}\right)}_{p}/{\left(C_{p}\right)}_{f}\right]+\left(1-\phi \right)\rho_{f}}{\rho_{\mathrm{nf}}} \end{array}$$

The corresponding initial and boundary conditions in nondimensional form are as follows:

t′≤0:u=θ=0,forxandy,

(17) $$t>0:\{\begin{array}{l} u=v=\theta =0,\;\mathrm{at}\;x=0,\\ u=v=0,\;\theta =1\;\mathrm{at}\;y=0,\;x>0,\\ u=0,\;\theta =0\;\;\mathrm{at}\;y\to \infty ,\;x>0 \end{array}$$

The quantities of physical interest, such as the local Nusselt number, average Nusselt number, local skin friction coefficient, and average skin friction coefficients are given as follows:

Local Nusselt number:

$$\mathrm{Nu}=\frac{x'h}{k_{f}}h=\frac{q_{w}}{\left(T_{w}^{'}-T_{\infty }^{'}\right)}q_{w}=-k_{\mathrm{eff}}{\left(\frac{\partial T'}{\partial y'}\right)}_{y'=0}.$$

Introducing nondimensional parameters defined in Equation 13, we get the following:

(18) $$\mathrm{Nu}=-x\frac{k_{\mathrm{eff}}}{k_{f}}{\left(\frac{\partial \theta }{\partial y}\right)}_{y=0}.$$

Similarly, the average Nusselt number in nondimensional form is as follows:

(19) $${\mathrm{Nu}}_{\mathrm{avg}}=-\frac{k_{\mathrm{eff}}}{k_{f}}\overset{1}{\underset{0}{\int }}{\left(\frac{\partial \theta }{\partial y}\right)}_{y=0}\mathrm{dx}.$$

The local skin friction coefficient in nondimensional form is as follows:

(20) $$\mathrm{Cf}=\frac{\mu_{\mathrm{nf}}}{\mu_{f}}{\left(\frac{\partial u}{\partial y}\right)}_{y=0}.$$

Average skin friction coefficient in non dimensional form:

(21) $${\mathrm{Cf}}_{\mathrm{avg}}\frac{\mu_{\mathrm{nf}}}{\mu_{f}}\overset{1}{\underset{0}{\int }}{\left(\frac{\partial u}{\partial y}\right)}_{y=0}\mathrm{dx}.$$

### Method of solution

In order to solve the nonlinear coupled partial differential equations (Equations 14, 15, and 16) along with the initial and boundary conditions (Equation 17), an implicit finite difference scheme for a three-dimensional mesh is used. The finite difference equations corresponding to these equations are as follows:

(22) $$\begin{array}{c} \frac{1}{4\mathrm{\Delta x}}\left(u_{i,j}^{n+1}-u_{i-1,j}^{n+1}+u_{i-1,j-1}^{n+1}+u_{i-1,j}^{n}\right)\\ \;+u_{i,j-1}^{n}-u_{i-1,j-1}^{n}\\ \;+\frac{1}{2\mathrm{\Delta y}}\left(v_{i,j}^{n+1}-v_{i,j-1}^{n+1}+v_{i,j}^{n}-v_{i,j-1}^{n}\right)=0, \end{array}$$

(23) $$\begin{array}{l} \frac{1}{\mathrm{\Delta t}}\left(u_{i,j}^{n+1}-u_{i,j}^{n}\right)+\frac{1}{2\mathrm{\Delta x}}u_{i,j}^{n}\left(u_{i,j}^{n+1}-u_{i-1,j}^{n+1}+u_{i,j}^{n}-u_{i-1,j}^{n}\right)\\ \;+\frac{1}{4\mathrm{\Delta y}}v_{i,j}^{n}\left(u_{i,j+1}^{n+1}-u_{i,j-1}^{n+1}+u_{i,j+1}^{n}-u_{i,j-1}^{n}\right)\\ \;=\frac{\mathrm{Pr}}{2{\left(\mathrm{\Delta y}\right)}^{2}}\frac{\mu_{c}}{\rho_{c}}\left(u_{i,j-1}^{n+1}-2u_{i,j}^{n+1}+u_{i,j+1}^{n+1}+u_{i,j-1}^{n}-2u_{i,j}^{n}+u_{i,j+1}^{n}\right)\\ \;+\beta_{c}\mathrm{Ra}\mathrm{Pr}\frac{1}{2}\left(\theta_{i,j}^{n+1}+\theta_{i,j}^{n}\right)\\ \;-\frac{\mathrm{Pr}}{\mathrm{Da}}\frac{\mu_{c}}{\rho_{c}}\frac{1}{2}\left(u_{i,j}^{n+1}+u_{i,j}^{n}\right)-\frac{\mathrm{Fr}}{\mathrm{Da}}\left|u_{i,j}^{n}\right|\frac{1}{2}\left(u_{i,j}^{n+1}+u_{i,j}^{n}\right) \end{array}$$

(24) $$\begin{array}{c} \frac{\sigma }{\mathrm{\Delta t}}\left(\theta_{i,j}^{n+1}-\theta_{i,j}^{n}\right)+\frac{1}{2\mathrm{\Delta x}}u_{i,j}^{n}\left(\theta_{i,j}^{n+1}-\theta_{i-1,j}^{n+1}+\theta_{i,j}^{n}-\theta_{i-1,j}^{n}\right)\\ \;+\frac{1}{4\mathrm{\Delta y}}v_{i,j}^{n}\left(\theta_{i,j+1}^{n+1}-\theta_{i,j-1}^{n+1}+\theta_{i,j+1}^{n}-\theta_{i,j-1}^{n}\right)\\ \;=\frac{k_{\mathrm{eff}}\left(\theta_{i,j}^{n}\right)}{\alpha_{f}{\left(\rho C_{p}\right)}_{\mathrm{nf}}}\left(\theta_{i,j-1}^{n+1}-2\theta_{i,j}^{n+1}+\theta_{i,j+1}^{n+1}+\theta_{i,j-1}^{n}-2\theta_{i,j}^{n}+\theta_{i,j+1}^{n}\right)\\ \;+\frac{A\left(\theta_{i,j}^{n}\right)}{\alpha_{f}{\left(\rho C_{p}\right)}_{\mathrm{nf}}}{\left(\frac{\theta_{i,j+1}^{n}-\theta_{i,j-1}^{n}}{2\mathrm{\Delta y}}\right)}^{2}\\ \;-\frac{\mathrm{Ec}}{C_{p_{c}}}\left|u_{i,j}^{n}\right|\left(\frac{u_{i,j-1}^{n}+u_{i,j+1}^{n}}{2}\right)\frac{\mathrm{Ec}}{C_{p_{c}}}u_{i,j}^{n}\\ \;\times \left[\mathrm{Pr}\frac{\mu_{c}}{\rho_{c}}\left(\frac{u_{i,j+1}^{n}-2u_{i,j}^{n}+u_{i,j-1}^{n}}{{\left(\mathrm{\Delta y}\right)}^{2}}-\frac{1}{\mathrm{Da}}\frac{u_{i,j-1}^{n}+u_{i,j+1}^{n}}{2}\right)\right], \end{array}$$

Equations 23 and 24 can be written in the following form:

(25) $$A_{1}u_{i-1,j}^{n+1}+B_{1}u_{i,j-1}^{n+1}+C_{1}u_{i,j-1}^{n+1}+D_{1}u_{i,j+1}^{n+1}=E_{1},$$

$$\begin{array}{c} A_{2}\theta_{i}{{}_{-1,j}}^{n}{}^{+1}+B_{2}\theta u_{i}{{}_{,j}}^{n}{}^{+1}+C_{2}\theta_{i}{{}_{,j-1}}^{n}{}^{+1}+D_{2}\theta_{i}{{}_{,j+1}}^{n}{}^{+1}\\ \;=E_{2}. \end{array}$$

Here, *A*_*i*_, *B*_*i*_, *C*_*i*_, *D*_*i*_, and *E*_*i*_ (*i* = 1, 2) in Equation 25 are constants for a particular value of *n*. The subscript *i* denotes the grid point along the *x* direction, *j* along the *y* direction, and *n* along the time (*t*) direction. The grid point (*x*, *y*, *t*) are given by (*i*Δ*x*, *j*Δ*y*, *n*Δ*t*)*.* In the considered region, *x* varies from 0 to 1 and *y* varies from 0 to *y*_max_. The value of *y*_max_ is 1.0, which lies very well outside the momentum and thermal boundary layers. Initially, at *t* = 0, all the values of *u*, *v*, and *T* are known. During any one time step, the values of *u* and *v* are known at previous time level. For every time step, first, the values of *T* are calculated using Equation 24, and then the values of *u* and *v* are calculated using Equations 23 and 22, respectively. At every time level, Equations 23 and 24 form a tridiagonal set of equations in the form of Equation 25. This tridiagonal system is solved by the Thomas algorithm, described by Carnahan et al. [27]. The solution of these equations is marched in time until the steady state is achieved. The steady-state solution is assumed to have been reached when the absolute difference between the values of *u* and *v* as well as the average value of the Nusselt number and average value of skin friction coefficient at two consecutive time steps is less than 10^−5^. The grid sizes are taken as *Δx* = 0.05, Δ*y* = 0.05, and Δ*t* = 6.25 × 10^−5^. Using Fourier expansion method and following Abd El-Naby et al. [28], it can be shown that the finite difference scheme described above is unconditionally stable and consistent. Therefore, the Lax-Richtmyer theorem implies convergence of the scheme [29]. We also checked the convergence of method using the computer code written in MATLAB to solve the above finite difference equations. The computer code was run for various grid spacing and various time intervals, and we found that if the grid spacing or the time spacing is further reduced, then there was no difference in the results. This shows that the scheme is convergent. To find the Nusselt number, skin friction coefficient, average Nusselt number, and average skin friction coefficient, the derivatives that appeared in Equations 18 to 21 are evaluated using the five point Newton's derivative formulae, and the definite integrations are evaluated using Simpson's integration formula.

### Validation of the formulation

To check the validity of formulation, we checked our results with some of the experimental as well as theoretical work done before. For this, we chose to study natural convection of water in glass bead porous media in the same conditions as the previous works had done. The parameters of porous media and the fluid and the results of calculations are given in Tables 1 and 2.

**Table 1:** **Nusselt number values for wall temperatures with permeability** = **1**.**2** × **10**^−**9**^**and 1**/**Da** = **3**.**375** × **10**^**6**^

**Plate temperature *T***_***w ***_ (**K**)	**RaK**	**Nu**	**Nu**_**avg**_	**Nu**/**RaK**^**0**.**5**^	**Nu**/**RaK**^**0**.**5**^**[**[1-4]**]**
333	235.7341	6.6866	11.1941	0.4355	≈0.44
353	353.6012	8.1777	13.1036	0.4349	0.44
373	471.4683	9.4101	14.5680	0.4344	0.44
393	589.3353	10.4920	15.7691	0.434	0.44

Diameter of glass bead (porous media) = 1 mm, length of plate = 0.1 m, permeability = 1.2 × 10^−9^, 1/Da = 3.375 × 10^6^, *T* (ambient) = 293 K.

**Table 2:** **Nusselt number values for wall temperatures with permeability** = **1**.**4683** × **10**^−**9**^**and 1**/**Da** = **2**.**8605** × **10**^**6**^

**Plate temperature *T***_***w ***_ (**K**)	**RaK**	**Nu**	**Nu**_**avg**_	**Nu**/**RaK**^**0**.**5**^	**Nu**/**RaK**^**0**.**5**^**[**[1-4]**]**
303	69.5325	3.6319	6.6569	0.4356	≈0.44
313	139.0649	5.0880	8.959	0.4315	0.44
323	208.5974	6.2634	10.5969	0.4337	0.44
333	278.1298	7.2200	11.8779	0.4329	0.44
353	417.2597	8.8231	13.8479	0.4320	0.44
373	556.2597	10.1248	15.3437	0.43	0.44

Diameter of glass bead (porous media) = 1 mm, length of plate = 0.1 m, permeability = 1.4683 × 10^−9^, 1/Da = 2.8605 × 10^6^, *T* (ambient) = 293 K.

**Figure 2:**
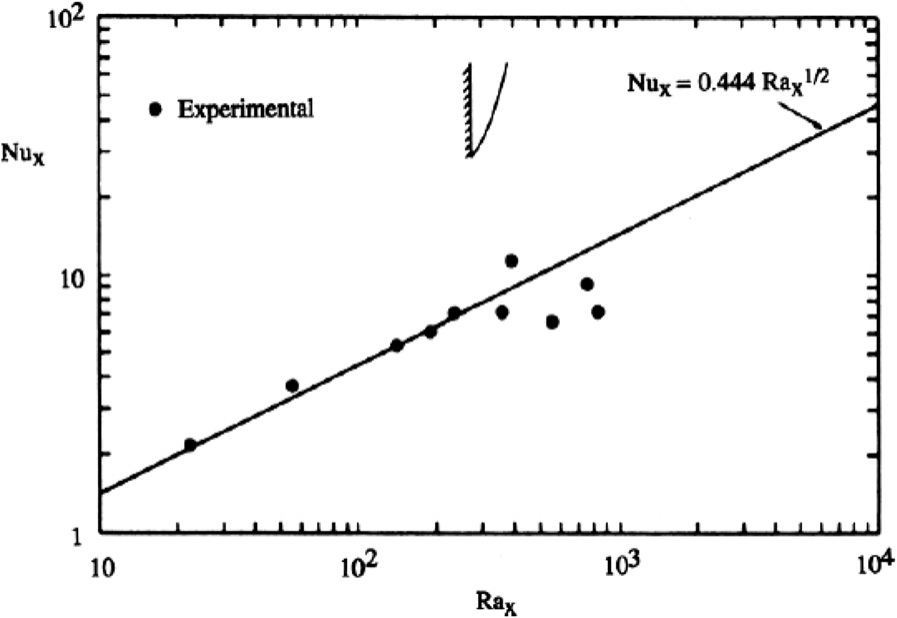
**Theoretical data from Cheng and Minkowycz[**[1]**]and experimental data from Evans and Plumb[**[2]**].** Graph adapted from Neild and Bejan [9].

First of all, we found the steady state for the flow. After finding the steady state, the values of the local Nusselt number for various values of the modified Rayleigh number ($\mathrm{RaK}=\frac{g\rho_{f}\beta_{f}\left(T_{w}^{'}-T_{\infty }^{'}\right)\mathrm{LK}}{\varepsilon \alpha_{f}\mu_{f}}$) have been calculated for different values of permeability of the medium containing glass spheres of 1 mm in diameter. These values are compared with the values found by some research (experimentally and theoretically) for the steady state. Cheng and Minkowycz [1] studied free convection about a vertical flat plate embedded in a porous medium for steady-state flow. They used the boundary layer approximations to get the similarity solution for the problem and found the value of the local Nusselt number Nu = 0.444 RaK^0.5^. Evans and Plumb [2] experimentally investigated the natural convection about a vertical plate embedded in a medium composed of glass beads with diameters ranging from 0.85 to 1.68 mm. Their experimental data were in good agreement with those of the theory of Cheng and Minkowycz [1] as shown in Figure 2. Hsu [4] and Kim and Vafai [5] showed that, in the case of an isothermal wall, the local Nussel number Nu = *C* × RaK^0.5^; here, *C* is a constant and depends upon the porous media and the fluid. These results for the steady-state natural convection of water in porous media have also been verified by various authors and can be found in the book by Neild and Bejan [9]. From our calculations given in Tables 1 and 2, it is clear that for various values of modified Rayleigh numbers, the value of Nu/RaK^0.5^ is almost constant, and the value of this constant is ≈ 0.44. This implies that our results are in good agreement with those of the work done previously.

## Results and discussion

Computations have been done for the vertical plate with a length of 40 mm placed in the copper powder (porous medium). The ambient temperature is considered to be 293 K. The value of Forchheimer coefficient (*F*) is taken as 0.55. Calculations have been done for six different types of nanofluids, *viz*. Al_2_O_3_ + H_2_O, TiO_2_ + H_2_O, CuO + H_2_O, Al_2_O_3_ + ethylene glycol (EG), TiO_2_ + EG, and CuO + EG, with different nanoparticle concentration and particle diameter in the temperature range of 293 to 324 K. Base fluid thermophysical properties are taken at the intermediate temperature, i.e., 308 K, to get a good correlation between thermal conductivity and viscosity data used by Corcione [14].

### Heat transfer enhancement at steady state using nanofluids

**Figure 3:**
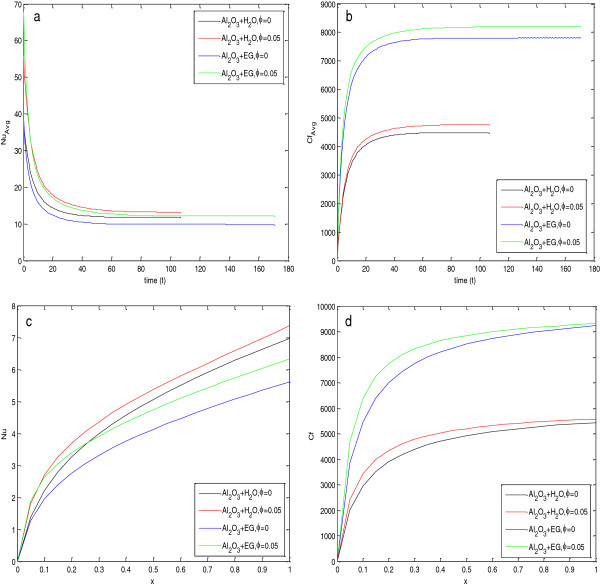
**Comparison between(a,b,c,d)Al**_**2**_**O**_**3**_+ **H**_**2**_**O and Al**_**2**_**O**_**3**_ + **EG at 324 K.**

To find the steady state of flow and heat transfer, the average Nusselt number and average skin friction coefficients are plotted with time, as show in Figure 3. From Figure 3a,b, it is observed that the average Nusselt number and average skin friction coefficient decrease very fast initially, but after a certain time, these values become constant. The constant behavior of the average Nusselt number and average skin friction coefficient guarantees the steady state. For water-based nanofluids, values of the average Nusselt number and average skin friction coefficients are constant after 100 s, i.e., steady state can be achieved after 100 s for water-based nanofluids. Similarly, for EG-based nanofluids, the steady state is achieved after nearly 160 s. This implies that the water-based nanofluids achieve a steady state earlier than the EG-based nanofluids. The reason for this behavior is the higher values of effective thermal diffusivity and lower values of volumetric heat capacity ratio of water-based nanofluids than EG-based nanofluids, as given in Table 3.

**Table 3:** Properties of six different types of nanofluids

**Nanofluid**	***α***_**eff**_**(10**^−**7**^**)**	***σ***	**Pr**_**eff**_	**RaK**_**eff**_	***μ***_**nf**_	**Nu**_**avg**_	**Cf**_**avg**_**(10**^**3**^**)**
Al_2_O_3_ + H_2_O	2.6100	0.9266	3.1656	101.6234	9.1980 × 10^−4^	13.1848	4.7330
TiO_2_ + H2O	2.5443	0.9234	3.2048	104.3849	9.1980 × 10^−4^	13.2042	4.7204
CuO + H_2_O	2.9179	0.9519	2.5879	91.3187	9.1980 × 10^−4^	12.5223	4.8192
Al_2_O_3_ + EG	1.8052	1.0160	73.4908	139.8607	1.6100 × 10^−2^	12.1085	8.1741
TiO_2_ + EG	1.7409	1.0096	75.2862	145.0326	1.6100 × 10^−2^	12.1394	8.1421
CuO + EG	2.1278	1.0711	57.4017	118.6878	1.6100 × 10^−2^	11.1641	8.3152

*ε* = 0.72, diameter of Cu powder = 470 μm, length of plate = 0.04 m, permeability = 7 × 10^−9^, *T* (ambient) = 293 K, *T*_*w*_ = 324 K, *d*_*p*_ = 10 nm, *ϕ* =0.04.

To find the percentage increase in heat transfer using nanofluids in porous media, two types of nanofluids have been used for calculations of the average Nusselt number and average skin friction coefficients at steady state, and the calculated values are compared with the case of pure fluid in porous media. The values of parameters taken in the calculations are given in Table 3. From Figure 3a and Table 4, it is clear that the value of the average Nusselt number at the steady state for the EG-based nanofluid is lesser than that of the water-based nanofluid, but the percentage increase in the value of the average Nusselt number is much more in the case of the EG-based nanofluid.

**Table 4:** **Average Nusselt number and average skin friction coefficients for Al**_**2**_**O**_**3**_ + **H**_**2**_**O and Al**_**2**_**O**_**3**_ + **EG**

**Nanofluid**	***Φ***	**Nu**_**avg**_	**Percentage increase in Nu**_**avg**_**at steady state**	**Cf**_**avg**_ (**10**^**3**^)	**Percentage increase in Cf**_**avg**_**at steady state**
Al_2_O_3_ + H_2_O	0	11.7178	12.11%	4.4865	6.34%
Al_2_O_3_ + H_2_O	0.05	13.1371		4.7711	
Al_2_O_3_ + EG	0	9.8380	23.16%	7.8077	5.06%
Al_2_O_3_ + EG	0.05	12.1162		8.2028	

*ε* = 0.72, diameter of Cu powder = 470 μm, length of plate = 0.04 m, permeability = 7 × 10^−9^, *T* (ambient) = 293 K, *T*_*w*_ = 324 K, *d*_*p*_ = 10 nm.

Figure 3c,d depicts the variation of local Nusselt number and local skin friction coefficients along the length of the plate at steady state. It is clear from Figure 3c that the local Nusselt number increases very fast along the plate up to a fixed distance, but after that, it increases almost linearly. The slope of this linearly increasing effect is larger for the water-based nanofluid as compared with the EG-based nanofluid. Figure 3d shows that the skin friction coefficients for the EG-based nanofluid is much larger than those for water-based nanofluid, and this resisted the motion of fluid, which is the reason why the Nusselt numbers for EG-based nanofluids are lesser than those of the water-based nanofluids.

### Temperature dependence of heat transfer enhancement and determination of optimal particle concentration in Al_2_O_3_ + water nanofluid

**Figure 4:**
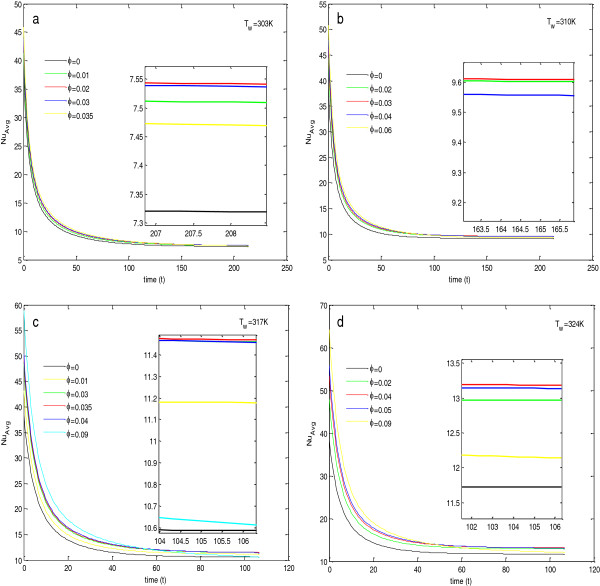
**Average Nusselt numbers for Al**_**2**_**O**_**3**_ + **H**_**2**_**O nanofluid at(a,b,c,d)different wall temperatures.**

**Figure 5:**
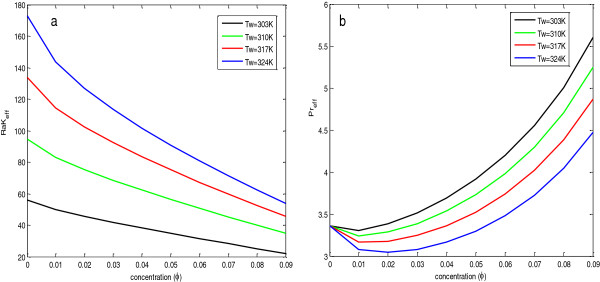
**Effective Prandtl number(a)and modified Rayleigh number(b)of Al**_**2**_**O**_**3**_ + **H**_**2**_**O nanofluid with concentration.**

**Figure 6:**
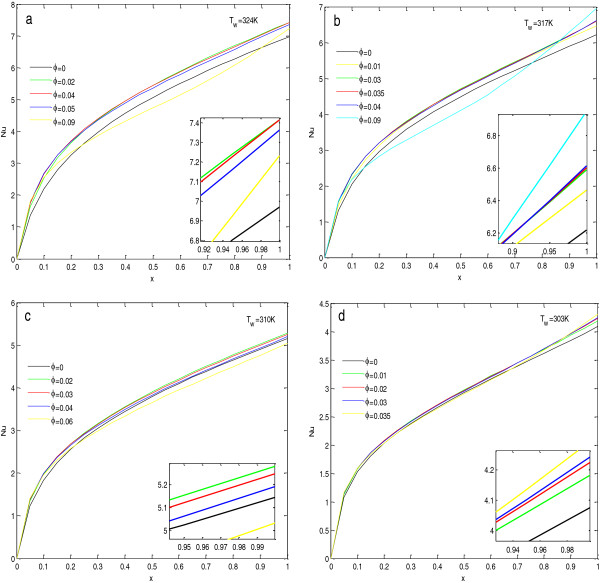
**Local Nusselt numbers for Al**_**2**_**O**_**3**_ + **H**_**2**_**O nanofluid at(a,b,c,d)different wall temperatures.**

**Figure 7:**
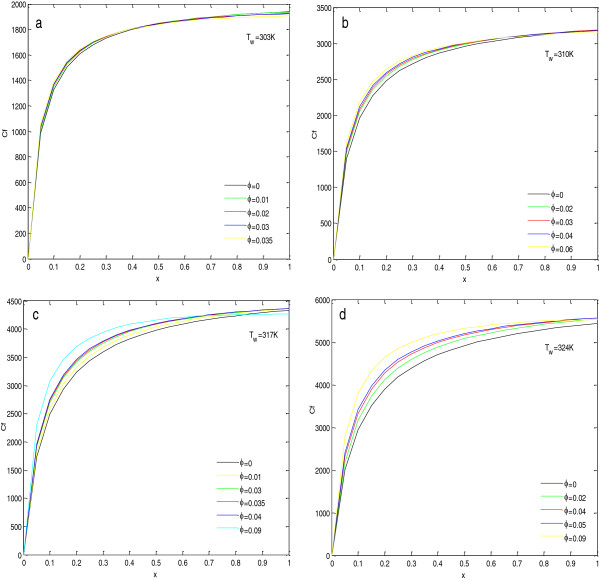
**Local skin friction coefficient for Al**_**2**_**O**_**3**_ + **H**_**2**_**O nanofluid at(a,b,c,d)different wall temperatures.**

To find the effect of concentration of nanoparticles in the base fluid, calculations have been done, and the results are shown in Figures 4, 5, 6, and 7 and given in Tables 5, 6, 7, and 8. In Figure 4, the insets show the zoomed view at steady state.

**Table 5:** Variation in average Nusselt number and average skin friction coefficient with concentration at 303 K

***Φ***	**Nu**_**avg**_	**Percentage increase in Nu**_**avg**_**at steady state**	**Cf**_**avg**_ (**10**^**3**^)	**Percentage increase in Cf**_**avg**_**at steady state**
0	7.3157	-	1.7009	-
0.01	7.5058	2.60	1.7150	0.36
*0*.*02*	*7*.*5363*	*3*.*02*	1.7154	0.38
0.025	7.5313	2.95	1.7150	0.36
0.04	7.4612	1.99	1.7130	0.24

*ε* = 0.72, diameter of Cu powder = 470 μm, length of plate = 0.04 m, permeability = 7 × 10^−9^, *T* (plate) = 303 K, *d*_*p*_ = 10 nm (Al_2_O_3_ + H_2_O).

**Table 6:** Variation in average Nusselt number and average skin friction coefficient with concentration at 310 K

***Φ***	**Nu**_**avg**_	**Percentage increase in Nu**_**avg**_**at steady state**	**Cf**_**avg**_ (**10**^**3**^)	**Percentage increase in Cf**_**avg**_**at steady state**
0	9.1505	-	2.7202	-
0.02	9.5864	4.76	2.7592	1.43
*0*.*03*	*9*.*5875*	*4*.*78*	2.7686	2.09
0.04	9.5262	4.11	2.7767	2.08
0.06	9.2465	1.05	2.7916	2.62

*ε* = 0.72, diameter of Cu powder = 470 μm, length of plate = 0.04 m, permeability = 7 × 10^−9^, *T* (plate) = 310 K, *d*_*p*_ = 11 nm (Al_2_O_3_ + H_2_O).

**Table 7:** Variation in average Nusselt number and average skin friction coefficient with concentration at 317 K

***Φ***	**Nu**_**avg**_	**Percentage increase in Nu**_**avg**_**at steady state**	**Cf**_**avg**_ (**10**^**3**^)	**Percentage increase in Cf**_**avg**_**at steady state**
0	10.5850	-	3.6357	-
0.01	11.1776	5.60	3.6945	1.62
0.02	11.3780	7.49	3.7244	2.44
0.03	11.4590	8.26	3.7483	3.10
*0*.*035*	*11*.*4674*	*8*.*34*	3.7589	3.39
0.04	11.4576	8.24	3.7690	3.67
0.06	11.2646	6.42	3.8052	4.66
0.09	10.6124	0.26	3.8493	5.88

ε = 0.72, diameter of Cu powder = 470 μm, length of plate = 0.04 m, permeability = 7 × 10^−9^, *T* (plate) = 317 K, *d*_*p*_ = 11 nm (Al_2_O_3_ + H_2_O).

**Table 8:** Variation in average Nusselt number and average skin friction coefficient with concentration at 324 K

***Φ***	**Nu**_**avg**_	**Percentage increase in Nu**_**avg**_**at steady state**	**Cf**_**avg**_ (**10**^**3**^)	**Percentage increase in Cf**_**avg**_**at steady state**
0	11.7178	-	4.4865	-
0.01	12.6260	7.75	4.5904	2.32
0.02	12.9659	10.65	4.6463	3.56
*0*.*04*	13.1848	12.52	4.7330	5.49
0.05	13.1371	12.11	4.7711	6.34
0.06	13.0020	10.96	4.8076	7.16
0.09	12.1363	3.57	4.9109	9.46

*ε* = 0.72, diameter of Cu powder = 470 μm, length of plate = 0.04 m, permeability = 7 × 10^−9^, *T* (plate) = 324 K, *d*_*p*_ = 11 nm (Al_2_O_3_ + H_2_O).

From Figure 4, it is depicted that for a particular value of concentration, the average Nusselt number decreases with time and attains a steady state after a particular time. At the start of heat flow, if we increase the concentration, the average Nusselt number is always higher than its value at lower concentration level, but as the process moves toward the steady state, the average Nusselt number decreases after a fixed concentration level, and this concentration level depends upon the temperature of the plate.

To analyze the effect of concentration at the steady state, the values of average Nusselt numbers and average skin friction coefficients at steady state have been found and given in Tables 5, 6, 7, and 8. From the tables, it is clear that the heat transfer rate and skin friction coefficient at the steady state are highly dependent on the wall temperature as well as the nanoparticle concentration in the base fluid. For a fixed wall temperature, the average Nusselt number first increases with the increase in nanoparticle concentration, but after a fixed concentration, it decreases with further increase in concentration. From Tables 5, 6, 7, and 8 and Figure 4, it is observed that this optimal concentration, for which the percentage increase in the average Nusselt number is maximum, depends on the wall temperature. As the wall temperature increases, the optimal concentration level of nanoparticle also increases. From these tables, it is also clear that the increase in wall temperature also increases the average Nusselt number. Therefore, for the maximum heat transfer rate, the temperature of the wall should be at its maximum along with the optimal particle concentration. The reason for these variations in Nusselt number values is justified by the fact that the Nusselt number depends upon the effective modified Rayleigh number and the Prandtl number of the fluid in porous media. From Table 9 and Figure 5a, it is clear that with the increase in concentration level, the modified Rayleigh number decreases, but with the increase in temperature, the modified Rayleigh number increases. Table 9 and Figure 5b depict that for a particular temperature and with the increase in concentration, the value of the Prandtl number decreases up to a particular concentration level, and then it increases. Also, with the increase in temperature, the minimum value of the Prandtl number shifts toward the higher value of concentration. Therefore, the Prandtl number and the effective modified Rayleigh numbers are responsible for the change in behavior with concentration and increase in value of the average Nusselt number with the increase in temperature, respectively.

**Table 9:** **Thermophysical properties of Al**_**2**_**O**_**3**_ + **H**_**2**_**O nanofluid for different wall temperatures and concentration**

	**Properties**	**Values**							
At *T* = 324 K, *d*_*p*_ = 10 nm, *ε* = 0.72	*Φ*	0	0.01	0.02	0.03	0.04	0.05	0.06	0.09
	*ρ* (10^3^)	0.998	1.0268	1.0556	1.0845	1.1133	1.1421	1.1709	1.2574
	*β*_nf_ (10^3^)	0.214	0.2062	0.1989	0.1919	0.1853	0.179	0.1731	0.1568
	*C*_pnf_ (10^3^)	4.187	4.1871	4.1872	4.1873	4.1874	4.1875	4.1876	4.1878
	*μ*_nf_	0.0007	0.0007	0.0008	0.0009	0.0009	0.001	0.0011	0.0016
	*k*_nf_	0.6288	0.7281	0.7857	0.8339	0.8768	0.9161	0.9529	1.0523
	*k*_eff_	0.8728	1.0106	1.0905	1.1573	1.2167	1.2713	1.3222	1.46
	*α*_eff_ (10^−6^)	0.2089	0.235	0.2467	0.2548	0.261	0.2658	0.2697	0.2773
	Pr_eff_	3.358	3.0766	3.0441	3.0801	3.1656	3.2973	3.4795	4.4709
	RaK_eff_	172.7511	143.4813	126.9420	113.4505	101.6234	90.8972	80.8972	53.9553
	*Σ*	0.9505	0.944	0.9379	0.9321	0.9266	0.9214	0.9164	0.9029
At *T* = 317, *d*_*p*_ = 10 nm	*Φ*	0	0.01	0.02	0.03	0.04	0.05	0.06	0.09
	*k*_nf_	0.6288	0.7079	0.7538	0.7922	0.8264	0.8577	0.887	0.9662
	*k*_eff_	0.8728	0.9826	1.0462	1.0995	1.1468	1.1903	1.2309	1.3407
	*α*_eff_ (10^−6^)	0.2089	0.2285	0.2367	0.2421	0.246	0.2489	0.251	0.2546
	Pr_eff_	3.358	3.1643	3.1728	3.242	3.3584	3.5216	3.7376	4.8687
	RaK_eff_	133.7428	114.2469	102.4320	92.4494	83.4695	75.1410	67.2753	45.4884
At *T* = 310 K, *d*_*p*_ = 10 nm	*Φ*	0	0.01	0.02	0.03	0.04	0.05	0.06	0.09
	*k*_nf_	0.6288	0.6915	0.7279	0.7584	0.7854	0.8103	0.8335	0.8963
	*k*_eff_	0.8728	0.9598	1.0103	1.0525	1.0901	1.1245	1.1567	1.2438
	*α*_eff_ (10^−6^)	0.2089	0.2232	0.2286	0.2318	0.2338	0.2351	0.2359	0.2362
	Pr_eff_	3.358	3.2393	3.2857	3.3867	3.5334	3.7276	3.9773	5.2482
	RaK_eff_	94.7345	82.8428	75.1371	68.4071	62.2040	56.3387	50.7101	34.7324
At *T* = 303, *d*_*p*_ = 10 nm	*Φ*	0	0.01	0.02	0.03	0.04	0.05	0.06	0.09
	*k*_nf_	0.6288	0.6783	0.707	0.731	0.7523	0.7719	0.7902	0.8398
	*k*_eff_	0.8728	0.9414	0.9812	1.0145	1.0441	1.0713	1.0967	1.1654
	*α*_eff_ (10^−6^)	0.2089	0.219	0.222	0.2234	0.224	0.224	0.2237	0.2213
	Pr_eff_	3.358	3.3026	3.383	3.5135	3.6888	3.9128	4.195	5.6014
	RaK_eff_	55.7261	49.6834	45.5077	41.7463	38.2001	34.7866	31.4619	21.8057

In Tables 5, 6, 7, and 8, the values of average skin friction are also given. It can be seen in the tables that the average skin friction coefficient always increases with the increase in particle concentration and wall temperature. This means that the increased concentration increases the viscosity of the nanofluid (as given in Table 9), and it hinders the motion of nanofluid particles and causes the increase in skin friction coefficients.

The effect of particle concentration on the local Nusselt number and local skin friction coefficients at steady state are shown in Figures 6 and 7. In these figures, the inset inside the figure shows the zoomed view near the end of the plate. From the figures, it is observed that with the increase in concentrations, the local Nusselt number always increases near the lower end of the plate, but this increase is very small. As we move upward along the plate, the local Nusselt number starts to decrease after the optimal concentration level. For very high concentrations (as compared to optimal concentration level), the local Nusselt number initially increases near the lower end of the plate, and then its value becomes the smallest, and near the upper end of the plate, it becomes the highest, as shown in Figure 6a,b. This abnormal behavior at high concentrations may be due to the increased nanoparticle clustering with the increase in concentration of nanoparticles in the base fluid.

Figure 7 depicts that with the increase in concentration of the nanoparticle in the base fluid, local skin friction coefficient increases. This is because of the increase in viscosity of the nanofluid with the increase in concentration as given in Table 9.

### Dependence on particle diameter

**Figure 8:**
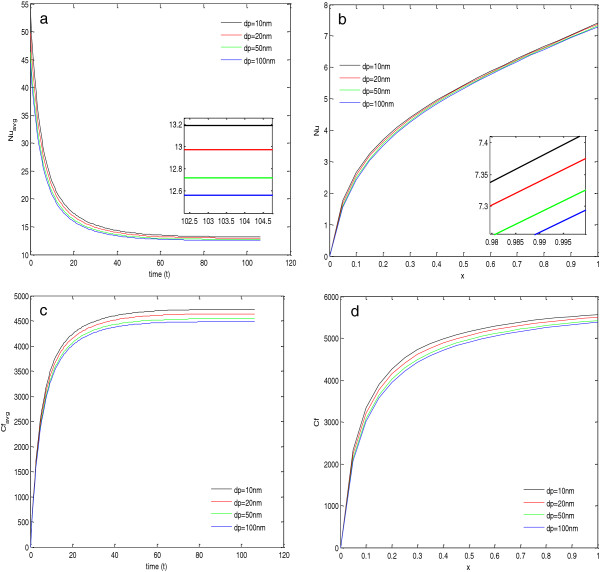
**Nusselt numbers and skin friction coefficients for(a,b,c,d)different particle diameters.**

In this section, the effect of nanoparticle size on heat transfer and skin friction coefficient for Al_2_O_3_+ H_2_O nanofluid is discussed. Here, all the calculations have been done at 324 K (wall temperature). Figure 8a,b depicts that the average Nusselt number as well as local Nusselt number both decrease with the increase in the size of nanoparticle. The reason for the deterioration in Nusselt number is the decreased thermal conductivity of the nanofluid with the increase in particle diameter. Similarly, the viscosity of the nanofluid decreases with the increase in particle diameter (given in Table 10); therefore, it decreases the skin friction coefficient. This effect of particle size on the skin friction can be seen in the Figure 8c,d. These figures show that the average skin friction coefficient as well as the local skin friction coefficient both decrease with the increase in particle size.

**Table 10:** **Properties of Al**_**2**_**O**_**3**_ + **H**_**2**_**O nanofluid for different particle diameters**

**Properties**	**Particle diameters *d***_***p***_ (**nm**)						
	**10**	**25**	**40**	**55**	**70**	**115**	**130**
*μ*_nf_(10^−3^)	0.9198	0.8553	0.831	0.8171	0.8077	0.7908	0.7871
*k*_nf_	0.8768	0.8007	0.7712	0.7542	0.7427	0.7222	0.7177
*k*_eff_	1.2167	1.1112	1.0703	1.0467	1.0307	1.0023	0.9961
*α*_eff_ (10^−6^)	0.261	0.2384	0.2296	0.2245	0.2211	0.215	0.2137
Pr_eff_	3.1656	3.2229	3.2511	3.2687	3.2812	3.304	3.309
RaK_eff_	101.6243	119.6707	127.8621	132.9777	136.6173	143.4837	145.0528

*T* = 324, *Φ* = 0.04, and *ε* = 0.72.

### Comparison between different nanofluids

**Figure 9:**
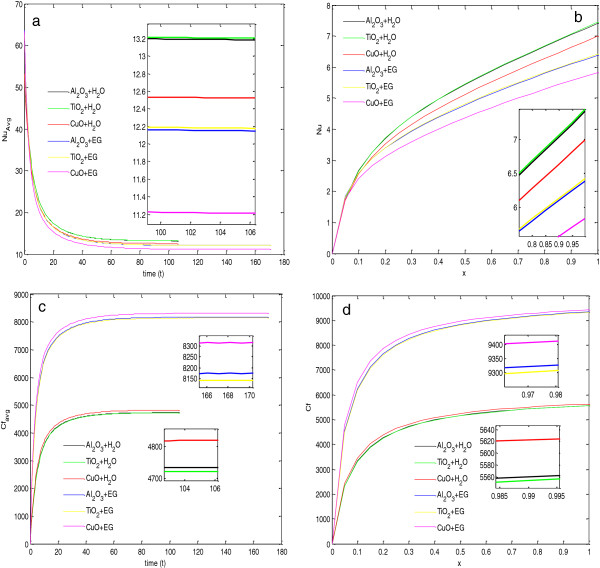
Comparison between six different types of nanofluids.

In this section, six types of nanofluids have been studied. The comparative study of different nanofluids is shown in Figure 9 and Table 3. In the previous section, it has been found that the optimal concentration for the Al_2_O_3_ + water nanofluid at 324 K wall temperature is 0.04, and for maximum heat transfer rate, the particle diameter should be minimum. Therefore, we used this value of concentration and the particle diameter of 10 nm. From Figure 9 and Table 3, it is concluded that there is only a small change in the value of Nusselt numbers (average and local) as well as skin friction coefficients for nanofluids having the same base liquid (H_2_O/EG). Among these values, the value of the average Nusselt number is in its maximum, in case of liquids containing TiO_2_. From Table 3, it is also clear that for the EG-based nanofluids, the value of effective RaK is larger than the water-based nanofluids, but still, the value of the average Nusselt number for water-based nanofluids is larger than that of EG-based nanofluids. It is because of the large difference in the values of skin friction coefficients. In the case of EG-based nanofluids, the average value of skin friction coefficient is almost double than the water-based nanofluids, which decreases the average Nusselt number. From this table, it can be verified that the increase in average Nusselt number is highly dependent on the nature of base liquid rather than the nature of the nanoparticle.

### Dependence on porosity and permeability of the medium

**Figure 10:**
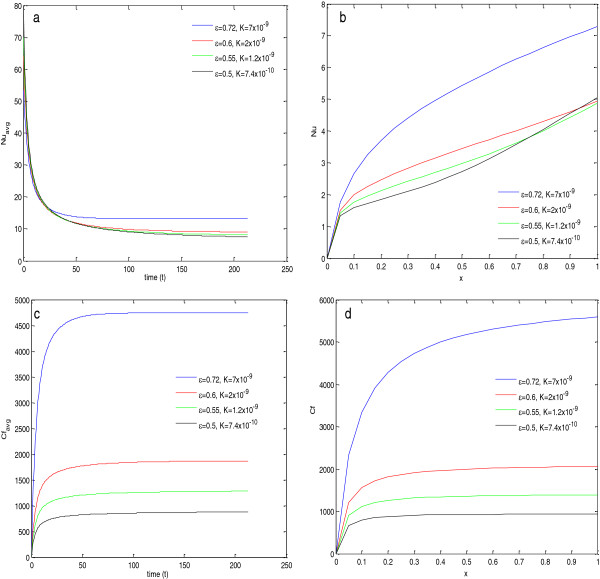
**Nusselt numbers and skin friction coefficients for different values of porosity of medium for Al**_**2**_**O**_**3**_ + **H**_**2**_**O at 324 K.**

The porosity and permeability effect of the medium on the Nusselt number and skin friction coefficient is shown in Figure 10. In the simulation, the radius of the copper powder (porous media) is kept constant, and the permeability of media has been calculated for different values of porosity using the relation $K=\frac{d_{\mathrm{eff}}^{2}\varepsilon^{3}}{150{\left(1-\varepsilon \right)}^{2}}.$

From this figure, it is clear that, as the porosity of the medium increases, the values of average Nusselt number, local Nusselt number, average skin friction coefficient, and local skin friction coefficient increase. The reason for the increase in Nusselt numbers with the increase in porosity is due to the major increase in RaK_eff_ with the increase in porosity, as given in Table 11. The reason for the increased skin friction coefficients can be explained with the help of the definition of porosity, where it is a measure of the void spaces in a material and is a fraction of the volume of voids over the total volume. Therefore, as porosity increases, the fraction of void space increases and results in the increase in roughness of the material, and hence, it increases the skin friction for the flow.

**Table 11:** Variation in physical properties with the porosity of medium

**Properties**	**Porosity *ε***			
	**0**.**5**	**0**.**55**	**0**.**6**	**0**.**72**
*K*	7.4 × 10^−10^	1.2 × 10^−9^	2 × 10^−9^	7 × 10^−9^
*k*_eff_	1.7497	1.59137	1.4592	1.2167
*α*_eff_ (10^−7^)	3.7534	3.4135	3.1301	2.6100
Pr_eff_	2.2013	2.4204	2.6396	3.1656
RaK_eff_	10.7041	17.5821	28.8800	101.7845
*σ*	0.8689	0.8820	0.8951	0.9266

*T* = 324, *Φ* =0.04, and *d*_*p*_ = 10 nm.

## Conclusions

In the present study, we have numerically investigated the natural convection heat transfer of nanofluids along the isothermal vertical plate embedded in a porous medium. For thermal conductivity and viscosity of nanofluids, empirical equations have been used, which suit best with the experimental data and incorporate the velocity-slip effects of the nanoparticles with respect to the base fluid. The effective thermal conductivity of the nanofluid in porous media has been taken into account. Here, three different nanoparticles, *viz*. Al_2_O_3_, CuO, and TiO_2_ with a valid range of particle concentration and particle size, have been taken with two base fluids, *viz*. water and EG. The natural convection of water in porous media had been initially studied, and we found a good agreement with the result available in the literature. The main findings of the study are as follows:

Percentage increase in the average Nusselt number at steady state for EG-based nanofluids is much more than that in the water-based nanofluids, and the percentage increase in average skin friction coefficient at steady state is almost the same in both cases.

The value of the average Nusselt number at steady state for water-based nanofluids is more than that of the EG-based nanofluids, but the value of the average skin friction coefficient at steady state for water-based nanofluids is much lesser than that of the EG-based nanofluids.

For the nanofluids with the same base fluid and different nanoparticles, there is a very small difference in the average Nusselt number and average skin friction coefficients. Among these values, the average Nusselt number and average skin friction coefficient for fluid containing TiO_2_ are a bit higher than those of the other two nanofluids.

From the three results, it is concluded that the heat transfer in nanofluids highly depends upon the nature of the base fluid rather than the nature of the added nanoparticles.

The average Nusselt number increases with the increase in nanoparticle concentration up to an optimal particle concentration and after it decreases. With the increase in plate temperature the optimal nanoparticle concentration level increases.

The average value of skin friction coefficient always increases with the increase in nanoparticle concentration.

For a particular value of concentration, the smallest nanoparticles enhance the heat transfer the most; skin friction coefficient also increases with the decrease in nanoparticle size.

For high values of porosity of the medium, the Nusselt number and skin friction coefficients are larger than their values in the low porosity medium.

In our future study, we will consider the effects of fouling and boiling in nanofluids and its effect on heat transfer. We will also perform some experiments for the natural convection of nanofluids in the same configuration and we will compare the numerical results with experiments.

## Nomenclature

*C*_*P*_: specific heat (J.kg^−1^.K^−1^); *d*: diameter (m); Da: Darcy number; Ec: Eckert number; *F*: Forchheimer's constant; Fr: Forchheimer's coefficient; *g*: gravitational acceleration (9.81 m.s^−2^); *K*: permeability (m^2^); *k*: thermal conductivity (W.m^−1^.K^−1^); *k*_*b*_: Boltzmann's constant **(**1.3806503 × 10^−23^ m^2^.kg.s^−2^.K^−1^); *L*: length of the plate (m); *M*: molecular weight of fluid (kg.mol^−1^); *N*: Avogadro's number (6.0223 × 10^23^); Pr: base fluid Prandtl number; Ra: Rayleigh number; RaK: modified Rayleigh number; Re: nanoparticle Reynold's number; *T′*: temperature (K); *u* and *v*: dimensionless velocities in the *x* and *y* directions; *u′* and *v′*: velocity component in the *x′* and *y′* direction (m.s^−1^).; *ρ*: Density (kg.m^−3^); *μ*: dynamic viscosity (Pa.s); *σ*: volumetric heat capacity ratio of medium; *ε*: porosity; *α*: thermal diffusivity (m^2^.s^−1^); *β*: coefficient of volume expansion (K^−1^); *θ*: dimensionless temperature; *Φ*: percentage of nanoparticle in base fluid.; ∞: Ambient fluid; avg: average; *c*: nondimensional coefficient; eff: effective property in porous medium; *f*: base fluid; *m*: porous medium; nf: nanofluid; *p*: nanoparticle; *w*: plate surface.

## Greek symbols

*C*_*P*_: specific heat (J.kg^−1^.K^−1^); *d*: diameter (m); Da: Darcy number; Ec: Eckert number; *F*: Forchheimer's constant; Fr: Forchheimer's coefficient; *g*: gravitational acceleration (9.81 m.s^−2^); *K*: permeability (m^2^); *k*: thermal conductivity (W.m^−1^.K^−1^); *k*_*b*_: Boltzmann's constant **(**1.3806503 × 10^−23^ m^2^.kg.s^−2^.K^−1^); *L*: length of the plate (m); *M*: molecular weight of fluid (kg.mol^−1^); *N*: Avogadro's number (6.0223 × 10^23^); Pr: base fluid Prandtl number; Ra: Rayleigh number; RaK: modified Rayleigh number; Re: nanoparticle Reynold's number; *T′*: temperature (K); *u* and *v*: dimensionless velocities in the *x* and *y* directions; *u′* and *v′*: velocity component in the *x′* and *y′* direction (m.s^−1^).; *ρ*: Density (kg.m^−3^); *μ*: dynamic viscosity (Pa.s); *σ*: volumetric heat capacity ratio of medium; *ε*: porosity; *α*: thermal diffusivity (m^2^.s^−1^); *β*: coefficient of volume expansion (K^−1^); *θ*: dimensionless temperature; *Φ*: percentage of nanoparticle in base fluid.; ∞: Ambient fluid; avg: average; *c*: nondimensional coefficient; eff: effective property in porous medium; *f*: base fluid; *m*: porous medium; nf: nanofluid; *p*: nanoparticle; *w*: plate surface.

## Subscripts

*C*_*P*_: specific heat (J.kg^−1^.K^−1^); *d*: diameter (m); Da: Darcy number; Ec: Eckert number; *F*: Forchheimer's constant; Fr: Forchheimer's coefficient; *g*: gravitational acceleration (9.81 m.s^−2^); *K*: permeability (m^2^); *k*: thermal conductivity (W.m^−1^.K^−1^); *k*_*b*_: Boltzmann's constant **(**1.3806503 × 10^−23^ m^2^.kg.s^−2^.K^−1^); *L*: length of the plate (m); *M*: molecular weight of fluid (kg.mol^−1^); *N*: Avogadro's number (6.0223 × 10^23^); Pr: base fluid Prandtl number; Ra: Rayleigh number; RaK: modified Rayleigh number; Re: nanoparticle Reynold's number; *T′*: temperature (K); *u* and *v*: dimensionless velocities in the *x* and *y* directions; *u′* and *v′*: velocity component in the *x′* and *y′* direction (m.s^−1^).; *ρ*: Density (kg.m^−3^); *μ*: dynamic viscosity (Pa.s); *σ*: volumetric heat capacity ratio of medium; *ε*: porosity; *α*: thermal diffusivity (m^2^.s^−1^); *β*: coefficient of volume expansion (K^−1^); *θ*: dimensionless temperature; *Φ*: percentage of nanoparticle in base fluid.; ∞: Ambient fluid; avg: average; *c*: nondimensional coefficient; eff: effective property in porous medium; *f*: base fluid; *m*: porous medium; nf: nanofluid; *p*: nanoparticle; *w*: plate surface.

## Competing interests

The authors declare that they have no competing interests.

## Authors’ contributions

ZU carried out the formulation and computation of the problem, found the results, and drafted the manuscript. SH read the manuscript and wrote the conclusion part of the paper. All authors read and approved the final manuscript.

## Authors’ information

ZU is a post doctoral researcher in the http://Université de Valenciennes et du Hainaut-Cambrésis, Valenciennes, France. He got his Ph.D. from G.B. Pant University of Agriculture and Technology, Pantnagar, India. After his Ph.D., he worked as an assistant professor of Mathematics in India. His current research interests cover analytical and numerical solutions of nonlinear problems arising in applied sciences and engineering phenomena related to fluid flow and thermal systems. SH is a professor and vice president of the University of Valenciennes & Hainaut Cambresis, France. She guided many Ph.D. students and successfully finished many industrial and scientific projects.
